# Multidisciplinary Approaches to Cardiovascular-Kidney-Metabolic Syndrome: Lessons from the Kidney-Heart Service

**DOI:** 10.34067/KID.0000001178

**Published:** 2026-04-30

**Authors:** Emeline Reniers, Pierre Letourneau

**Affiliations:** Department of Nephrology, Hospices Civils de Lyon, Centre Hospitalier Lyon-Sud, Pierre-Bénite, France

**Keywords:** cardiovascular disease, cardiovascular-kidney-metabolic (CKM) syndrome, cardiorenal syndrome

## Shift from Organ-Specific to an Integrated Cardiovascular-Kidney-Metabolic Care Model

The clinical management of patients with concomitant heart and kidney dysfunction has historically been fragmented. While cardiology traditionally focuses on hemodynamics, cardiac output, and afterload reduction, nephrology concentrates on glomerular filtration, electrolyte homeostasis, and volume status; these disciplines often operate in isolation.

However, mounting evidence demonstrates that cardiac disease and renal failure are inextricably linked: Major cardiovascular events frequently lead to CKD,^1^ while CKD itself is a potent cardiovascular risk factor.^2^ This interplay has led to the emergence of the cardiovascular-kidney-metabolic (CKM) syndrome framework,^3^ as defined by the American Heart Association. This paradigm shift necessitates a move toward an integrated, systems-based approach where the patient is viewed not as a collection of discrete pathologies, but as a complex system characterized by intertwined circulatory and metabolic failures.

The study by de Wolski *et al*.^4^ in this issue evaluates the University of Washington's dedicated Kidney-Heart Service (KHS). This model transitions from traditional reactive consultation to a proactive multidisciplinary service that prioritizes patients admitted to cardiology, cardiothoracic, and intensive care units. In its first year, the KHS launched several key initiatives: the development of streamlined communication strategies between nephrology and cardiology services, mandatory training of all attendings in point-of-care ultrasound (POCUS) for routine clinical decision-making, and the collaborative update and promotion of an evidence-based diuretic algorithm designed to improve efficacy and reduce dialysis requirements. This evolution mirrors shifts in other subspecialties, such as onco-nephrology, where the nephrologist must integrate specialty-specific knowledge (such as the nephrotoxicity of novel chemotherapies, management of monoclonal gammopathy of renal significance, or the effect of clonal hematopoiesis of indeterminate potential) to optimize renal outcomes. In the KHS model, the nephrologist acts as a cardiorenal specialist, applying renal physiological expertise to the broader context of systemic circulatory failure.

## Specialized Management of Mechanical Circulatory Support and Renal Perfusion

A hallmark of the cohort described by de Wolski *et al*. is its clinical complexity. With 22% of patients requiring mechanical circulatory support—including extracorporeal membrane oxygenation, ventricular assist devices, and percutaneous microaxial flow pumps like Impella. Management in this setting requires expertise that extends significantly beyond traditional nephrology training.

In the presence of mechanical circulatory support, traditional markers of renal function and hemodynamic stability are often altered. For example, continuous-flow ventricular assist devices change the pulsatility of renal blood flow, which may affect the renin-angiotensin-aldosterone system activation and intrarenal hemodynamics in ways that are not fully captured by standard monitoring. A dedicated service allows for the application of advanced physiological principles to navigate these challenges, including the use of POCUS for lung assessment and the objective measurement of venous congestion through venous excess ultrasound scoring.^5^ This tool enables the clinician to differentiate between absolute systemic hypervolemia and severe venous hypertension (backpressure). In the latter, even without an overall excess of fluid, the transmission of cardiac filling pressures to the renal veins creates a congestive gradient that opposes glomerular filtration, leading to congestive AKI.

Furthermore, the KHS model facilitates the implementation of standardized diuretic protocols. Sequential nephron blockade—combining loop diuretics with thiazides,^6^ carbonic anhydrase inhibitors (acetazolamide),^7^ or sodium-glucose transport 2 inhibitors^8^—is essential for overcoming the diuretic resistance frequently encountered in advanced heart failure. In selected cases, the controlled use of hypertonic saline in conjunction with high-dose loop diuretics may be used to recruit interstitial fluid and maintain intravascular volume for effective filtration.^9^ By focusing on the interplay between cardiac output, venous congestion, and renal perfusion pressure, a specialized service can mitigate AKI severity even in the most unstable patients. This multimodal management is particularly crucial for younger patients with CKD, who often face a disproportionate cardiovascular burden because of premature vascular aging.^10^

## The KHS Experience: Outcomes and Resource Utilization

The population managed by the KHS is characterized by high baseline mortality and a frequent requirement for KRT, reflecting the clinical severity in advanced CKM syndrome. In analyzing the KHS experience, de Wolski *et al*. compared outcomes against a historical control cohort in the same center. However, as with any descriptive study using historical data, significant caveats regarding causal inference must be acknowledged. Potential selection bias, residual confounding, temporal shifts in cardiac surgical techniques or medical treatment availability, shorter time to nephrology referral, and data collection methods mean that the observed differences may not represent a direct causal effect of the service implementation alone, as the authors correctly acknowledged.

Despite these methodological limitations, the results suggest a favorable trend in clinical markers. The study reports a reduction in in-hospital mortality (16.5% in the KHS cohort versus 25% in the historical control, *P* < 0.01) and a lower incidence of AKI requiring dialysis (36.7% versus 42%, *P* = 0.05). These improvements are noteworthy given the high-risk nature of the population and may be attributed to better management—such as shorter time to referral and standardized diuretic protocols with sequential nephron blockade—that permit less dialysis initiation and, consequently, lower mortality.

Conversely, these improved survival outcomes were accompanied by an increased length of stay (14 days for KHS versus 11 days for historical patients). This finding likely reflects a competitive risk effect: As more of the most critically ill patients survive the acute phase, they require an extended period of hospitalization for stabilization. In this context, the extra days spent in the hospital should not be viewed merely as a resource burden but as a necessary investment.

## The Challenge of Care Transition and Sustainable Models

Despite inpatient successes, de Wolski *et al*. highlight a vulnerability in the continuum of care: Only 1.7% of non–dialysis-dependent patients had nephrology follow-up within 60 days in the University of Washington system. While the authors acknowledged potential under-ascertainment for care delivered outside the University of Washington's system, it could nonetheless underscore a significant break in the care continuum. The transition from a highly monitored environment to outpatient care is a period of high risk. These patients possess an increasingly narrow therapeutic window, often oscillating rapidly between hypovolemia and congestion. Managing this vicious cycle requires frequent medical evaluations, sometimes monthly or even more often, to adjust complex drug regimens. As the prevalence of CKM syndrome rises, tertiary centers will likely be unable to absorb the entire volume of these patients. Therefore, the challenge is two-fold: first, the need to train community-based physicians in the specific management of these complex circulatory profiles and second, the development of sustainable care models. In regions with limited medical density, the integration of specialized nurse practitioners working in conjunction with telemedicine and remote monitoring may offer a viable solution. Such a decentralized system could provide the close physiological stewardship necessary to maintain stability and prevent rehospitalization without saturating specialized hospital services.

## Conclusion: Advancing the Standard of Care in Nephro-Cardiology

The experience of the University of Washington's KHS demonstrates that a specialized approach is suited to the management of high-complexity patients. The observed trends in mortality and dialysis requirements support the broader implementation of this model.

As CKM syndrome becomes more prevalent, clinical practice must evolve toward an integrated approach. Incorporating advanced hemodynamic monitoring, POCUS, and specialized pharmacological strategies allows clinicians to manage the multisystem instability inherent to this population.

Ultimately, the professional background of the specialist—whether originating from cardiology or nephrology—is of secondary importance to their ability to operate at this interface. Effective management of CKM syndrome requires practitioners capable of integrating hemodynamic data, venous congestion assessment, and complex pharmacology. This hybridized expertise represents a necessary evolution in clinical care, but the challenge will lie in the ability to extend this model beyond the hospital; without a scalable and sustainable outpatient system, the gains of the acute phase remain fragile.
